# Fatty Liver Index (FLI) Identifies Not Only Individuals with Liver Steatosis but Also at High Cardiometabolic Risk

**DOI:** 10.3390/ijms241914651

**Published:** 2023-09-27

**Authors:** Fabrizia Carli, Silvia Sabatini, Melania Gaggini, Anna Maria Sironi, Giorgio Bedogni, Amalia Gastaldelli

**Affiliations:** 1 Cardiometabolic Risk Unit, Institute of Clinical Physiology, National Research Council (CNR), Via Giuseppe Moruzzi, 1, 56124 Pisa, Italy; fcarli@ifc.cnr.it (F.C.); silvia.sabatini2@student.unisi.it (S.S.); melania.gaggini@cnr.it (M.G.); sironi.am@gmail.com (A.M.S.); 2 Department of Medical and Surgical Sciences, University of Bologna, Via Zamboni, 33, 40126 Bologna, Italy

**Keywords:** MASLD, epicardial fat, cardiac fat, visceral fat, insulin resistance, metabolic syndrome, cardiometabolic risk

## Abstract

A fatty liver index (FLI) greater than sixty (FLI ≥ 60) is an established score for metabolic dysfunction-associated steatotic liver disease (MASLD), which carries a high risk for diabetes and cardiovascular disease, while a FLI ≤ 20 rules out the presence of steatosis. Thus, we investigated whether FLI was associated with cardiometabolic risk factors, i.e., visceral (VAT), subcutaneous (SC), epicardial (EPI), extrapericardial (PERI), and total cardiac (CARD-AT) adipose tissue, hepatic fat ((by magnetic resonance imaging, MRI, and spectroscopy, MRS), and insulin resistance (IR, HOMA-IR and OGIS-index), and components of metabolic syndrome. All individuals with FLI ≥ 60 had MASLD, while none with FLI ≤ 20 had steatosis (by MRS). Subjects with FLI ≥ 60 had a higher BMI and visceral and cardiac fat (VAT > 1.7 kg, CARD-AT > 0.2 kg). FLI was positively associated with increased cardiac and visceral fat and components of metabolic syndrome. FLI, VAT, and CARD-AT were all associated with IR, increased blood pressure, cholesterol, and reduced HDL. For FLI ≥ 60, the cut-off values for fat depots and laboratory measures were estimated. In conclusion, FLI ≥ 60 identified not only subjects with steatosis but also those with IR, abdominal and cardiac fat accumulation, increased blood pressure, and hyperlipidemia, i.e., those at higher risk of cardiometabolic diseases. Targeted reduction of FLI components would help reduce cardiometabolic risk.

## 1. Introduction

Cardiometabolic risk (CMR) refers to risk factors that increase the likelihood of developing diabetes or cardiovascular disease (CVD). Specific factors that can cause this increased risk include obesity (especially abdominal fat accumulation), hyperglycemia, hypertension, insulin resistance, dyslipoproteinemia, and, with respect to metabolic syndrome, include physical inactivity and smoking. Individuals with intra-abdominal (e.g., visceral, hepatic, and pancreatic) and/or intra-thoracic (as epicardial, mediastinal, and/or intramyocardial) fat have increased cardiometabolic risk [1,2,3,4], and this phenotype may be present even in subjects with a low BMI and normal weight that not only have abdominal obesity and/or hepatic steatosis but also have abnormal metabolism and increased cardiometabolic risk [4,5,6,7,8].

The metabolic origin of hepatic steatosis is now also recognized in the name of the disease. In fact, the American Association for Study of Liver Disease (AASLD) and the European Association for Study of the Liver (EASL), in collaboration with the Asociación Latino-americana para el Estudio del Hígado (ALEH), have changed the nomenclature of nonalcoholic fatty liver disease (NAFLD), which is now called metabolic dysfunction-associated steatotic liver disease (MASLD) and metabolic dysfunction-associated steatohepatitis (MASH) [9]. The nomenclature is still new, but the discrepancy between MASLD and NAFLD is minimal [10].

The definition of MASLD includes subjects with hepatic steatosis and at least one of five cardiometabolic risk factors, thus taking into consideration the pathophysiology of the disease that is related to insulin resistance and adipose tissue dysfunction [11,12]. When subcutaneous adipocytes enlarge to store excess energy intake, the subject is less likely to accumulate ectopic fat and develop MASLD [6,11,12], whereas if subcutaneous fat does not expand, excess fat accumulates as intra-abdominal fat (e.g., visceral, hepatic, and pancreatic) and/or intra-thoracic fat (as epicardial, mediastinal, and/or intramyocardial fat) [11,13,14,15,16]. This latter phenotype is associated with increased cardiometabolic risk [1,2,3,4].

The latest estimates indicate that over 1/3 of the population has MASLD with a rapid increase in prevalence from 25% to 38% in the latest years [17]. However, most of these individuals are unaware of their condition and the increased risk of comorbidities associated with it. This is because MASLD is usually diagnosed incidentally after an imaging test or during a liver biopsy for suspected liver disease. Some of the guidelines-approved scores for the estimation of liver steatosis could be used for an initial diagnosis [18]. Among these scores, the fatty liver index (FLI) developed by Bedogni et al. [19] is an established index to diagnose hepatic steatosis and thus MASLD [18], and one of the most widely used and validated worldwide [20,21,22,23,24]. The parameters used to calculate FLI, i.e., BMI, waist circumference (WC), triglycerides (TG), and γ-GT concentration, are also markers of increased risk of cardiometabolic disease [25,26,27,28,29,30]. In particular, WC is a surrogate marker of upper body obesity and abdominal fat accumulation, and a high γ-GT concentration is associated with atherosclerosis, cardiovascular risk and mortality [26,29,30,31,32,33,34,35,36,37,38].

Thus, the aim of this paper was to investigate if FLI > 60, which is the cut off to diagnose hepatic steatosis, could also identify subjects with increased visceral and cardiac fat, as well as with increased cardiometabolic risk given by insulin resistance and other components of metabolic syndrome such as high blood pressure and hyperlipidemia.

## 2. Results

Table 1 reports the clinical characteristics of the study subjects. Most of the subjects (83%) were men with a median age of 53 years (IQR 42–59). The body mass index (BMI) ranged from 19 to 40 kg/m^2^; obesity was present in 24% of them (*n* = 22); 21 of them had type 2 diabetes (T2D, i.e., a fasting plasma glucose ≥ 7.0 mmol/L or a 2 h plasma glucose ≥ 11.1 mmol/L), which was newly diagnosed in 13 of them by OGTT.

Table 1 also shows the characteristics of the 33 subjects who had liver fat measured by magnetic resonance spectroscopy (MRS). This subgroup had characteristics similar to those of the whole group.

**Figure 1:**
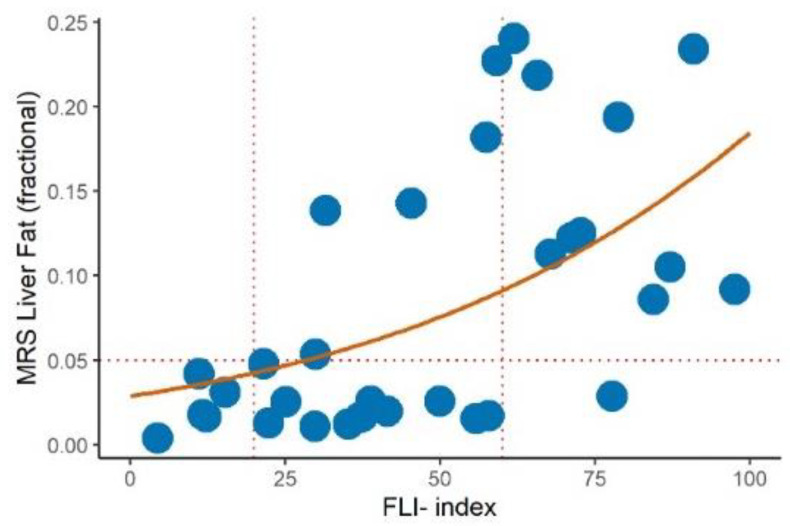
Relationship (beta regression with logit link) between the MRS liver fat fraction and fatty liver index (solid red line) in a subgroup of 33 subjects (blue dots). A cut off of IHTG > 5% (horizontal dashed line) was used to diagnose hepatic steatosis. Vertical dashed lines indicate fatty liver index (FLI) cut off of 20 and 60.

As can be seen from Figure 1, all subjects with a FLI ≥ 60 except one had a liver fat fraction IHTG > 0.05 (5%) that is the threshold for hepatic steatosis [18], confirming that a value of the FLI ≥ 60 was a good cutoff for the presence of hepatic steatosis according to the present operational definition. Using beta regression, we estimated the hepatic fat fraction to be 0.04 (95% CI 0.02 to 0.06) in the subjects with FLI = 20 and 0.09 (0.07 to 0.11) in those with FLI = 60. This corresponded to a difference of 0.05 (0.03 to 0.07) in the liver fat fraction between the two groups (*p* < 0.001).

**Figure 2:**
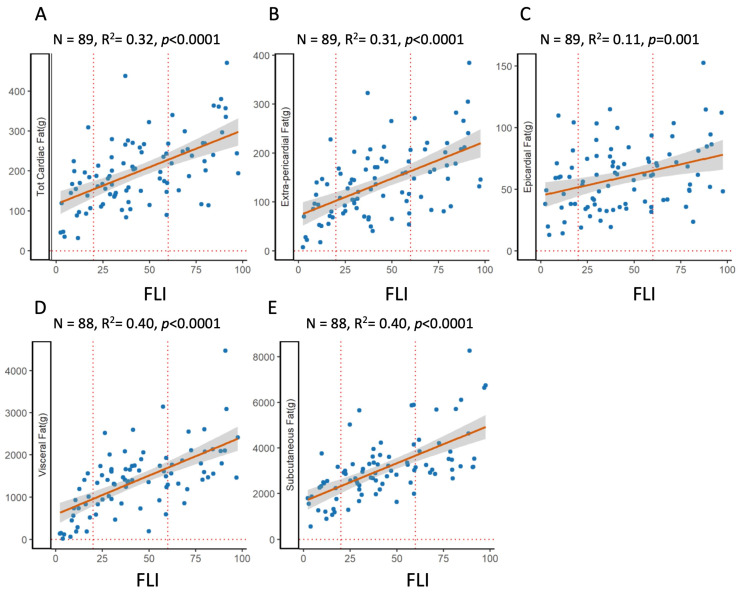
Relationship between fat depots and fatty liver index. OLS regression plots the association between FLI and various fat depots. **Top**: total cardiac fat (**A**), i.e., the sum of extra-pericardial (**B**) and epicardial (**C**) adipose tissue; **bottom**: visceral (**D**) and subcutaneous (**E**).

There was a strong positive association between FLI and visceral, subcutaneous, or cardiac adipose tissue, as shown in Figure 2. FLI explained 40% of the variance of both visceral and subcutaneous adipose tissue. The association of FLI with total cardiac adipose tissue was also strong and explained 32% of the variance; it is interesting that the variance was almost entirely explained by extra-pericardial fat.

**Figure 3:**
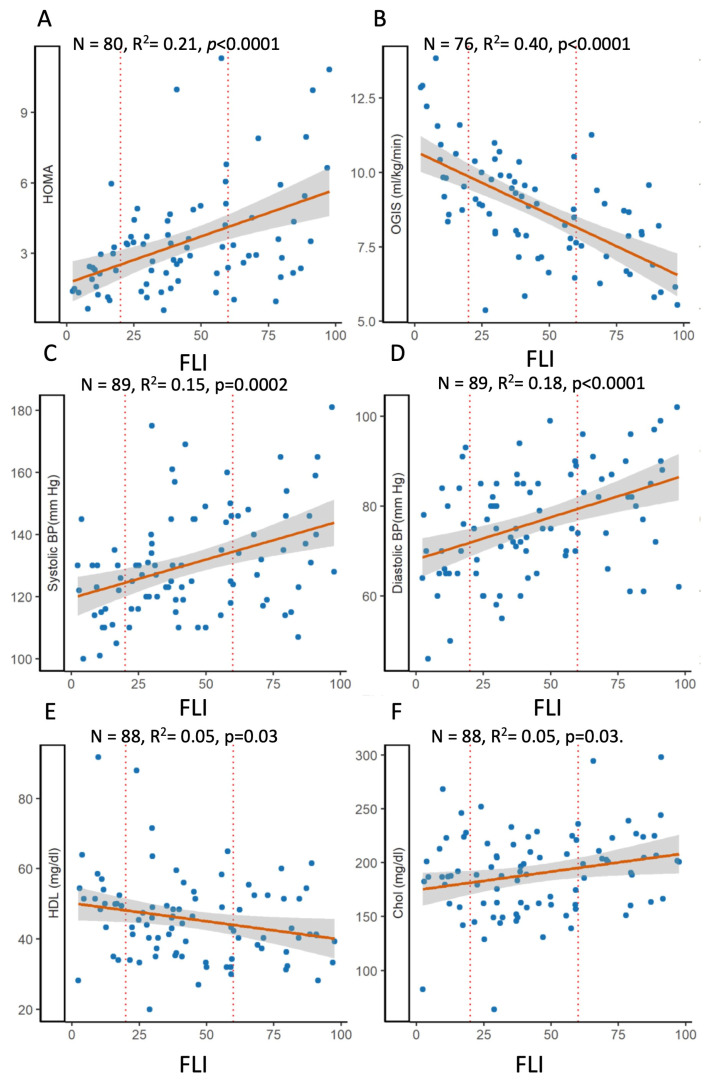
Relationship between fatty liver index and components of insulin resistance, HOMA-IR (**A**) and OGIS (**B**) or of the metabolic syndrome, i.e., systolic (**C**) and diastolic (**D**) blood pressure, HDL (**E**), and total cholesterol (**F**). OLS regression plots the association between the selected laboratory measures and FLI.

FLI was also strongly associated with insulin resistance (i.e., positive with HOMA-IR and negative with OGIS, which is an index of insulin sensitivity), but it was the insulin resistance measured in postprandial conditions (i.e., OGIS) and reflected mainly muscle IR that explained a great part of the variability, 40% (Figure 3). Also, blood pressure, in particular high diastolic blood pressure, was associated with the FLI, while the association with cholesterol, total and HDL, was modest, explaining only 5% of the variance.

Moreover, as shown in Table 2, not only the FLI but also VAT and CARD-AT were associated with IR, positively with HOMA-IR (Pearson coefficient *ρ* = 0.46, 0.49 and 0.31, respectively, all *p* < 0.005) and negatively with OGIS (*ρ* = −0.63, −0.64 and −0.48, respectively, all *p* < 0.0001), with increased diastolic blood pressure (*ρ* = 0.42, 0.32 and 0.32, respectively, all *p* < 0.0005), and reduced HDL (*ρ* = −0.23, −0.23 and −0.20, respectively, all *p* < 0.03). VAT and CARD-AT were also correlated with increased triglycerides (*ρ* = 0.45, and 0.39, respectively, all *p* < 0.0001).

Then, it was investigated which values of fat depots and laboratory measurements corresponded to the cut-off FLI values for ruling out or diagnosing hepatic steatosis, i.e., a FLI equal to 20 and 60, respectively (Table 3). To estimate these values, regression models were used as described under Statistical Analysis. Although this should be validated in other cohorts, the results indicated that a FLI ≥ 60 may be used to identify individuals at high cardiometabolic risk, and a FLI < 20 for those at low risk.

## 3. Discussion

The prevalence of obesity and related comorbidities continues to increase [39], and, along with obesity, MASLD is also on the rise. The latest estimates indicate that the prevalence of MASLD has increased from 25% (1990–2006) to 38% (2016–2019) [17]. Furthermore, the prevalence of cardiometabolic disease is increasing due to the steady rise of the prevalence of obesity worldwide [39], where obesity is defined as a body mass index (BMI) > 30 kg/m^2^. However, it is now evident that BMI alone does not fully capture the risk of disease or death [40], nor does it indicate how fat is distributed, i.e., the accumulation of visceral adipose tissue [41], epicardial and extrapericardial adipose tissue [26,42,43,44,45,46], or hepatic steatosis [47], which are established risk factors for cardiometabolic disease stronger than the BMI in both men and women [46]. MASLD is a cardiometabolic risk factor [7,8,48,49], and subjects with MASLD have high visceral adipose tissue (VAT) [47] and cardiac fat [50] compared to those of subjects without steatosis, even when BMI is below 25 kg/m^2^ [34,47].

Thus, we aimed to investigate whether FLI ≥ 60, which is the cut off for diagnosis of hepatic steatosis, could also identify subjects with increased visceral and cardiac fat, insulin resistance, and components of metabolic syndrome such as hypertension and hyperlipidemia, thus with high risk of cardiometabolic disease.

The results of our study showed that FLI was not only a marker of hepatic fat content (measured by MRS) but was also linearly correlated with increased visceral and cardiac fat, insulin resistance, particularly when measured during OGTT as the OGIS index, with increased blood pressure, and with total and HDL cholesterol, although less strongly (Table 2). Other studies have shown that individuals with increased visceral fat accumulation also have liver steatosis [51,52,53] and cardiac fat accumulation [49,54,55,56], but none using the FLI. In general, this reflects a condition of lipotoxicity and altered lipid metabolism [11,12] and explains why ectopic fat accumulation is an important risk factor for the development of cardiometabolic diseases such as diabetes, hypertension, metabolic syndrome, and cardiovascular disease [1,26,27,44].

Here, we have shown a strong linear association not only of the FLI but also visceral and cardiac fat with increased fasting insulin resistance (HOMA-IR) and with reduced glucose clearance (OGIS) during OGTT, which is an index of insulin sensitivity. This confirms previous results that showed an association between the severity of MASLD and insulin resistance measured during fasting conditions as HOMA-IR [57,58], during OGTT as Matsuda or OGIS index [58,59,60], or during a clamp [26,61].

It is recognized that adipose tissue is an active and complex organ with important endocrine functions and adverse metabolic consequences [6,11,62]. Adipose tissue accumulates primarily as subcutaneous fat, but when the individual becomes obese, he or she may accumulate a significant amount of fat as visceral or intrathoracic fat. Increased abdominal fat is also associated with the release of proinflammatory adipokines, the concentration of which is associated with elevated cardiometabolic risk [6].

It is well established that accumulation of abdominal fat, rather than total fat, is a major cause of the high prevalence of type 2 diabetes and cardiovascular disease [1]. Visceral adipose tissue has been associated with insulin resistance, hypertension, diabetes mellitus, and other metabolic abnormalities [1,47,51,52,53,63]. Increased VAT correlates with increased serum concentrations of small and dense LDL, low HDL, inflammatory markers, proinflammatory adipocytokine production, impaired insulin sensitivity, dyslipidemia and hypertension, and increased risk of endothelial dysfunction and thrombosis [1,49].

Hepatic steatosis is also associated with an increased risk of cardiovascular diseases [44,49]. This is not surprising, since these individuals have increased VAT [47], which is highly lipolytic [64] and releases free fatty acids (FFA) directly into the portal vein, making the liver the first organ where they are taken up before reaching systemic circulation [11]. Moreover, VAT amount is strongly correlated with hepatic fat accumulation [11,47], and the presence of high IHTG and VAT synergistically increases insulin resistance in the liver, muscles, and adipose tissue [44,49,53].

Moreover, the fat accumulated around the heart (as epicardial or mediastinal/extrapericardial fat) or within cardiomyocytes as lipid droplets is associated with metabolic abnormalities such as insulin resistance, increased serum TG and blood pressure, atherosclerosis, and altered cardiac function [3,43,65]. Epicardial fat is a potential key mediator of cardiac metabolism since FFA and adipokines released from these adipocytes can be taken up by coronary arteries and reach the heart [66]. Moreover, these cytokines promote macrophage differentiation in the intima, with the development of a pro-inflammatory environment that can lead to the development of coronary atherosclerosis [67].

The strength of this study lies in the extensive metabolic characterization of the subjects and the measurement of visceral, cardiac, and hepatic fat during the same exam, together with a measurement of insulin resistance during fasting and OGTT. We defined values for fat depots and laboratory measures for FLI values of 20 and 60 that could be used as cut-off or reference values (Table 3). Our study has some limitations, the most important of which is the relatively low number of subjects, and that it is not a longitudinal study. However, the results are in agreement with previous analyses that found that the FLI is associated with an increased risk of cardiovascular disease in the UK Biobank cohort [68], type 2 diabetes (T2D) [27,69], atherosclerosis [26,45], chronic kidney disease [70,71,72], and mortality [38]. The FLI was developed in the general population with only mild obesity [19], although it has been widely used in different cohorts. Although our study included a limited number of subjects, to the best of our knowledge, there are no other studies that looked at the association between the FLI, visceral fat, and cardiac fat in the same group. The ability to identify individuals with increased VAT and CARD-AT is important, but the measurement requires MRI or computed tomography. In contrast, the FLI is easy to calculate and relatively inexpensive because it uses variables that are usually collected during routine clinical examination such as the BMI, waist circumference, and TG and GGT. Thus, the result of this study confirm the ability of the FLI to identify subjects at risk of cardiometabolic risk in line with previous studies that also showed how a FLI > 60 is able to identify the hazard of atherosclerosis and endothelial dysfunction, both at a subclinical stage and as an overt disease [26,29,30,31,32,33,34,35,36,37], and to detect incident diabetes [27,29,73,74,75] and chronic kidney disease [70,71,72].

In conclusion, the FLI can be used not only to identify individuals with MASLD but also those at higher risk of metabolic disorders underlying an increased risk of cardiovascular events, selecting those who would need to improve their lifestyle and possibly be referred to a specialist for further investigation. Its use may be broader, i.e., not only in primary care, but also secondary and tertiary care centers.

## 4. Materials and Methods

### 4.1. Study Protocol

This analysis included individuals who participated in previous protocols at the CNR Institute of Clinical Physiology in Pisa for measurement of abdominal and cardiac adipose tissue by magnetic resonance imaging (MRI) and spectroscopy (MRS). The subjects who had waist circumference, BMI, triglyceride, and gamma GT concentrations measured to calculate the fatty liver index (FLI) were included in this analysis (n = 89). A subgroup of subjects also had measurements of liver fat (n = 33). In 81 individuals, an oral glucose tolerance test (OGTT) was performed in the morning after an overnight fast (10–12 h) for the measurement of glucose tolerance and assessment of insulin sensitivity by the OGIS index [76]. Timed blood samples (at −15, 0, 30, 60, 90, 120 min) were collected for the measurement of plasma glucose (Beckman Glucose Analyzer, Fullerton, CA, USA) and insulin concentrations (Linco Research, St. Louis, MO, USA). Liver enzymes and serum lipid profile were determined by standard laboratory methods (Beckman Coulter AU400, Brea, CA, USA).

The study protocol was approved by the local Ethics Committee, and all individuals gave their informed consent to participate.

### 4.2. Magnetic Resonance (MRI-MRS) Study

We used magnetic resonance imaging (MRI) to evaluate cardiac and visceral fat, and magnetic resonance spectroscopy (MRS) to measure intrahepatic triglycerides (IHTGs) as the hepatic fat fraction.

The protocol has already been described [3]. Briefly, MRI acquisition of the heart was performed using a standardized protocol. Cardiac adipose tissue scans were obtained by fast-spin echo T1-weighted sequences with an oblique axial orientation using a cardiac coil and an ECG trigger. During the acquisition time, the patients were in breath-hold (10–12 s). Pericardial and epicardial fat areas were measured in a four-chamber view using an in-house semi-automated program to determine the margin of fat around the heart, identifying the region of interest (ROI) and measuring the number of pixels, as previously described [63]. We used the previously validated conversion factor of 0.076 g/mm^2^ [3] to calculate cardiac adipose tissue mass from area measurements.

Two fat depots could be easily distinguished: (a) epicardial adipose tissue (EPI), i.e., the fat concentrated in the atrioventricular and interventricular grooves, along the major branches of the coronary arteries, and, to a lesser extent, around the atria, over the free wall of the right ventricle and over the apex of the left ventricle (LV); (b) pericardial adipose tissue (PERI), i.e., the fat situated on the external surface of the parietal pericardium within the mediastinum, also termed mediastinal or intrathoracic fat [63]. Visceral and subcutaneous fat images were acquired during the same MRI session using imaging procedures published previously [63]. Briefly, 32 transverse, T1-weighted images centered through the space between L4 and L5 were acquired in breath hold. Abdominal visceral (VAT) and subcutaneous fat depots (SC) were quantified using imaging procedures and in house ad hoc developed software, “Hippo fat software” (2.0) [3]. A factor of 0.92 kg/cm^3^ was used to convert adipose tissue volume into adipose tissue mass [63].

In a subgroup of 33 subjects, we also measured intrahepatic triglycerides by water suppression ^1^H-MRS using the single-voxel stimulated acquisition mode (STEAM) (TR/TE = 5000/12 ms) with a GE body coil as previously described [52]. Typical voxel size was 3.5 mm × 3.5 mm × 3.5 mm, placed within the right lobe of the liver, avoiding the vessels, bile ducts, and focal lesions. The quantification was achieved using NUTS software (1D version, Acorn NMR Inc., Livermore, CA, USA) after correcting the T1 and T2 relaxation times as previously described [77]. A cut off of 5% was used to rule out hepatic steatosis according to European guidelines [18].

### 4.3. Calculations and Statistical Analysis

We calculated several insulin resistance (IR) indexes [78]. HOMA-IR was calculated as (fasting glucose (mmol/L) × insulin (mU/L))/22.5. A value of HOMA-IR > 2 was used as the cut-off since it was found to be associated with hepatic fat above the normal values (i.e., >5.6%) [78]. We also calculated the OGIS index from glucose and insulin concentrations measured during OGTT, performed in 76 individuals [76]; OGIS is an index of insulin sensitivity as it measures glucose clearance, but it is also associated with the severity of MASLD, especially liver fibrosis [59,60].

The FLI (fatty liver index) was calculated using a previously described algorithm [19] based on BMI, waist circumference, TG, and GGT. The score varied between 0 and 100. With an accuracy of 0.84 (95% confidence interval (CI) 0.81–0.87) in detecting fatty liver, the FLI does not discriminate by sex or ethnicity and is calculated using waist circumference (WC in cm), body mass index (BMI), triglyceride concentration (TG in mg/dL), and GGT concentration (in mg/dL) as follow:

FLI = e^z^/(1 + e^z^)

where

z = 0.953 × ln(TG) + 0.139 × BMI + 0.718 × ln(GGT) + 0.053 × WC − 15.745

The cut off of FLI > 60 indicates a likelihood > 78% of the presence of steatosis; FLI < 20—a likelihood >91% of the absence of steatosis [28].

Thus, three groups with different risk were identified [28]: Group 1 = FLI ≤ 20 with a probability not to have MASLD >90%; Group 2 = FLI: 21–59, intermediate group; Group 3 = FLI ≥ 60 with a probability to have MASLD >78%.

Most continuous variables were not Gaussian-distributed, and all are reported as the median (50th percentile) and interquartile ranges (IQR, 25th and 75th percentiles). Discrete variables are reported as the number and proportion of individuals with the characteristic of interest.

The relationship between the liver fat fraction and the FLI was evaluated using beta regression with a logit link and robust confidence intervals in a subsample of 33 individuals. The outcome variable of the beta regression model was the liver fat fraction, and the predictor was the FLI [79]. The relationship between the outcome and the predictor was linear as detected by fractional polynomials [80]. Using the regression equation, we calculated the marginal estimates of the liver fat fraction at values of the FLI of 20 and 60 and contrasted them using the delta method [81]. The association between MRI-detected fat and laboratory measures and the FLI was evaluated using ordinary least square (OLS) regression in the full sample of 89 individuals. The outcome variable of the regression model was the fat compartment or laboratory measure, and the predictor was the FLI. We used fractional polynomials to test whether the outcome–predictor relationship was linear and found it to be so in all cases [80,81]. Statistical analysis was performed using Stata 15.1 (Stata Corporation, College Station, TX, USA).

## Figures and Tables

**Table 1:** Clinical characteristics of the study subjects.

Characteristics	Entire Cohort	Group with MRS
Number	89	33
Age (years)	53 (42; 59)	54 (42; 63)
Sex F/M	15 (17%)/74 (83%)	7 (21%)/26 (79%)
Weight (kg)	82.3 (71.7; 90.0)	83.0 (75.0–89.8)
Height (m)	1.74 (1.67; 1.79)	1.75 (1.67–1.80)
BMI (kg/m^2^)	27.1 (25.0; 29.9)	27.7 (25.5–29.4)
BMI class (NIH)		
Normal	22 (25%)	8 (24%)
Overweight	45 (51%)	17 (52%)
Obesity class 1	19 (21%)	7 (21%)
Obesity class 2	2 (2%)	1 (3%)
Obesity class 3	1 (1%)	-
Waist circumference (cm)	95.0 (89.0; 101.5)	96.0 (92.0–102.0)
ALT (U/L)	20 (17; 32)	24 (16–34)
AST (U/L)	21 (19; 25)	21 (19–26)
GGT (U/L)	24 (16; 36)	25 (17–42)
NGT/IGT/T2D	49/25/15	18/7/8
Glucose (mg/dL)	100 (92.5; 110.5)	100 (91–111)
Insulin (mU/mL)	12 (8.2; 17.1)	10 (7–17)
HOMA-IR	2.9 (2.0; 4.4)	2.6 (1.6–3.7)
QUICKI	0.17 (0.16; 0.18)	0.17 (0.16–0.19)
OGIS (mL/kg/min)	8.81 (7.68; 9.87)	9.19 (7.91–10.35)
Cholesterol (mg/dL)	190 (162; 216)	189 (162–211)
HDL (mg/dL)	44 (37; 51)	49 (40–54)
LDL (mg/dL)	123 (97; 138)	120 (96–136)
Triglycerides (mg/dL)	84 (67; 118)	87 (70–128)
Systolic BP (mm Hg)	128 (118; 142)	127 (117–140)
Diastolic BP (mm Hg)	75 (67; 85)	73 (65–85)
Fatty liver index (FLI)	39 (23; 64)	45 (30–68)
FLI category < 20	19 (21%)	5 (15%)
FLI category 20 to 59	45 (51%)	17 (52%)
FLI category ≥ 60	25 (28%)	11 (33%)
Liver fat (%)—MRS	4 (2–13)	4 (2–13)
Subcutaneous fat (g)—MRI	2970 (2326; 3590)	3079.6 (2460.2–4077.2)
Visceral fat (g)—MRI	1405 (873; 1761)	1407.5 (892.8–1830.4)
Epicardial fat (g)—MRI	60 (38; 76)	50 (35–72)
Extra-pericardial fat (g)—MRI	136 (82; 183)	112 (81–166)
Mediastinal fat (g)—MRI	187 (141; 252)	187 (117–235)

Measurements of the study subjects. The data are reported as the median (interquartile range) for continuous measures and number (proportion) for discrete measures. Some values were missing at random, e.g., for broken test tube.

**Table 2:** Associations between cardiometabolic parameters, FLI, VAT, and CARD-AT.

Outcome	FLI	VAT	CARD-AT
Total cardiac fat (g)	0.56 §	0.57 §	-
Extrapericardial fat (g)	0.55 §	0.57 §	0.96 §
Epicardial fat (g)	0.33 §	0.32 *	0.65 §
Visceral fat (g)	0.64 §	-	0.57 §
Subcutaneous fat (g)	0.63 §	0.34 *	0.33 *
HOMA-IR	0.46 §	0.49 §	0.31 *
OGIS	−0.63 §	−0.64 §	−0.48 §
Systolic BP (mm Hg)	0.39 §	0.33 *	0.37 §
Diastolic BP (mm Hg)	0.42 §	0.32 *	0.32 *
HDL (mg/dL)	0.23 *	0.17	0.10
Cholesterol (mg/dL)	−0.23 *	−0.23 *	−0.20
Triglyceride (mg/dL)	-	0.45 §	0.39 §

Pearson coefficient, * *p* < 0.05, § *p* < 0.001.

**Table 3:** Estimated reference values for fat depots and laboratory measures for FLI values of 20 and 60.

Outcome	FLI = 20	FLI = 60	*p*-Value
Hepatic fat	0.04 (0.04)	0.09 (0.04)	<0.001
Total cardiac fat (g)	154.37 (10.44)	228.53 (9.95)	<0.001
Extrapericardial fat (g)	102.64 (8.77)	163.20 (7.51)	<0.001
Epicardial fat (g)	51.73 (3.69)	65.33 (3.15)	0.02
Visceral fat (g)	958.82 (85.24)	1694.43 (74.15)	<0.001
Subcutaneous fat (g)	2329.96 (156.61)	3666.31 (136.24)	<0.001
HOMA-IR	2.53 (0.31)	4.12 (0.27)	<0.001
OGIS	9.87 (0.22)	8.16 (0.19)	<0.001
Systolic BP (mm Hg)	124.53 (2.27)	134.48 (1.94)	<0.001
Diastolic BP (mm Hg)	71.88 (1.57)	79.39 (1.34)	0.154
HDL (mg/dL)	48.17 (1.72)	44.02 (1.45)	<0.001
Cholesterol (mg/dL)	181.49 (5.55)	195.18 (4.68)	0.01

Median and interquartile range of values estimated from the underling regression models as described under Statistical Analysis.

## Data Availability

Data available upon reasonable request.
